# Long-term projections of the impacts of warming temperatures on Zika and dengue risk in four Brazilian cities using a temperature-dependent basic reproduction number

**DOI:** 10.1371/journal.pntd.0010839

**Published:** 2023-04-27

**Authors:** Hannah Van Wyk, Joseph N. S. Eisenberg, Andrew F. Brouwer

**Affiliations:** Department of Epidemiology, University of Michigan, Ann Arbor, Michigan, United States of America; California Department of Public Health, UNITED STATES

## Abstract

For vector-borne diseases the basic reproduction number $R_{0}$, a measure of a disease’s epidemic potential, is highly temperature-dependent. Recent work characterizing these temperature dependencies has highlighted how climate change may impact geographic disease spread. We extend this prior work by examining how newly emerging diseases, like Zika, will be impacted by specific future climate change scenarios in four diverse regions of Brazil, a country that has been profoundly impacted by Zika. We estimated a $R_{0}\left(T\right)$, derived from a compartmental transmission model, characterizing Zika (and, for comparison, dengue) transmission potential as a function of temperature-dependent biological parameters specific to *Aedes aegypti*. We obtained historical temperature data for the five-year period 2015–2019 and projections for 2045–2049 by fitting cubic spline interpolations to data from simulated atmospheric data provided by the CMIP-6 project (specifically, generated by the GFDL-ESM4 model), which provides projections under four Shared Socioeconomic Pathways (SSP). These four SSP scenarios correspond to varying levels of climate change severity. We applied this approach to four Brazilian cities (Manaus, Recife, Rio de Janeiro, and São Paulo) that represent diverse climatic regions. Our model predicts that the $R_{0}\left(T\right)$ for Zika peaks at 2.7 around 30°C, while for dengue it peaks at 6.8 around 31°C. We find that the epidemic potential of Zika will increase beyond current levels in Brazil in all of the climate scenarios. For Manaus, we predict that the annual $R_{0}$ range will increase from 2.1–2.5, to 2.3–2.7, for Recife we project an increase from 0.4–1.9 to 0.6–2.3, for Rio de Janeiro from 0–1.9 to 0–2.3, and for São Paulo from 0–0.3 to 0–0.7. As Zika immunity wanes and temperatures increase, there will be increasing epidemic potential and longer transmission seasons, especially in regions where transmission is currently marginal. Surveillance systems should be implemented and sustained for early detection.

## Author summary

Rising temperatures through climate change are expected to increase arboviral disease pressure, so understanding the impact of climate change on newly emerging diseases such as Zika is essential to prepare for future outbreaks. However, because disease transmission may be less effective at very high temperatures, it is uncertain whether risk will uniformly increase in different regions. Given the nonlinear relationship between temperature and many important biological vector traits, mathematical modeling is a useful tool for predicting the impact of temperature on arbovirus risk. We used a temperature-dependent infectious disease transmission model to derive a temperature-dependent basic reproduction number. We then used historical temperature data and temperature projections for the years 2045–2049 to forecast Zika risk in four cities in Brazil under various climate change scenarios. We predict an overall increase in arbovirus risk, as well as extended risk seasons in cities that are not currently suitable for year-round spread, such as Rio de Janeiro. We also found little-to-no protective effect of increasing temperatures even in warmer climates like Manaus. Our results indicate that preparation for future Zika outbreaks (and of those of other arboviruses including dengue) should include the implementation of national disease surveillance and early detection systems.

## Introduction

The Zika and dengue viruses are closely related arboviruses that are primarily transmitted to humans through the *Aedes aegypti* and *A. albopictus* mosquitoes. Brazil carries an especially large share of the disease burden, with an estimated 1.5 million Zika cases since the beginning of the 2015–16 outbreak [1]. Zika was introduced in the Americas in 2015 [2], causing numerous outbreaks in countries throughout Latin America, including Brazil, Colombia, and Venezuela. Because vector-borne disease transmission depends on temperature, recent work has outlined the potential for climate change to facilitate its re-emergence (and emergence in new regions) [3–5]. Given the concerning health outcomes of Zika—including microcephaly and Guillain-Barre syndrome—the unpredictability of how the changing climate will influence the spread of the virus throughout the Western hemisphere is a growing cause of concern.

Dengue has a longer history in the region, originally emerging in the Americas in the 1600s [6]. It was eliminated by the 1960s through widespread use of pesticides, but it re-emerged in the early 1980s [7]. Since its re-emergence, dengue has remained endemic throughout many Latin American countries [8]. Due to dengue’s endemicity and wide geographic spread, it has been better studied than Zika and provides a useful point of comparison as we consider the potential impact of climate change on these arboviruses.

As a result of climate change, it is estimated that about half of the world’s population will live in geographic regions that will be suitable for arbovirus transmission by the year 2050 [9]. Several factors make Brazil particularly vulnerable to both the drivers and impacts of climate change. Primary among these is deforestation within the Amazonian region, as well as widespread increases in temperature, both of which are conducive to mosquito breeding [10]. Arbovirus outbreaks, such as the Zika outbreak in Brazil in 2015, have also been attributed in part to El Niño conditions that year [11]; *Aedes aegypti*, the primary vector of Zika and dengue [12], is particularly suited to warm, humid conditions. Brazil, therefore, is an important region to study Zika transmission potential, as has been highlighted by global Zika projections [3, 5].

In the 1990s, when researchers started using mathematical modeling to consider the impacts of climate change on vector-borne disease transmission, several studies began to incorporate temperature-dependent parameters such as vector competence, vector lifespan, and extrinsic incubation period [5, 13]. Temperature impacts specific biological vector traits, including lifespan, vector competence, and extrinsic incubation period, through several mechanisms relating to viral and vector physiology (e.g., impacts on metabolic rates) [14, 15]. Previous empirical and modeling work has suggested that these various temperature dependencies combine in a such a way that disease risk increases with temperature to a maximum at an optimal temperature and decreases thereafter [16, 17]. More recently, temperature-dependent $R_{0}$s for vector-borne diseases have revealed an interesting range of peak temperatures depending on the pathogen and mosquito species [18–20]. Of particular interest, Mordecai et al. found that disease risk peaks at the highest temperatures for pathogens that are transmitted by the *A. aegypti* mosquito [20]. The same group has also theorized that this finding means that with increasing temperatures, vector-borne disease risk in Africa will shift from malaria to arboviral diseases [21]. As climate change has the potential to shift much of the world into temperatures where these higher peaks occur, it is important to better understand both the range of uncertainty across climate change scenarios as well as the likely geographic and temporal heterogeneity in disease risk.

We extend prior work [22] that developed temperature dependent $R_{0}$ expressions to forecast future global trends of Zika and, as a comparison, dengue transmission risk in Brazil for the years 2045–2049, across a range of plausible climate change scenarios. Specifically, we explore how projections might vary across regions within a country and the likely impact of year-to-year temperature variation. We developed a basic reproduction number $R_{0}\left(T\right)$ as a function of temperature-dependent vector parameters specific to *Aedes aegypti*, which we used to project seasonal disease risk in four Brazilian cities representative of the different climate regions of Brazil. Our work extends and complements previous temperature-dependent projections of arbovirus risk [3, 18–22] by assessing geographic and year-to-year heterogeneity in projected risk across climate change scenarios.

## Materials and methods

### Data

To examine the potential impacts of climate change across a variety of climates, we selected four cities representative of diverse climatic regions of Brazil: Manaus, a city in the Amazon Rainforest with a tropical rainforest climate; Recife, an Atlantic coastal city with a tropical monsoon climate; Rio de Janeiro, an Atlantic coastal city with a tropical savanna climate; and São Paulo, a southern city with a humid subtropical climate. All cities are at approximately sea level and within the suitable elevation range for an abundant *A. aegypti* population, i.e., up to 1,600 meters [23, 24].

We obtained the historical and projected future temperature data from ISIMIP (The Inter-Sectoral Impact Model Intercomparison Project) [25]. Specifically, we use the downscaled and bias-adjusted GFDL-ESM4 (Geophysical Fluid Dynamics Laboratory, NOAA) model [26–28]. We chose to use GFDL-ESM4 because it is the CMIP-6 (Coupled Model Intercomparison Project, phase 6) model generally agreed to most accurately capture historical temperatures in South America [29, 30]. Air temperature data was obtained using the daily-mean 2-meter air temperature variable. To extract temperature data for each of the four cities, we calculated the nearest model grid point to each city’s location, which is available at a 0.5°x0.5° latitude-longitude spatial resolution.

For our historical baseline, we used data from 2015–2019, a five-year period encompassing the Zika outbreak in Brazil. For our forecast, we used 30-year projections, i.e., projected temperature data for the years 2045–2049. For the temperature projections, we use four SSP (Shared Socioeconomic Pathways) climate scenarios: SSP126, SSP245, SSP370, and SSP585 [31]. These scenarios represent different climate-relevant levels of socioeconomic development (taking into consideration factors like sustainable consumption, protection of vulnerable land, fertility rates) and their corresponding greenhouse gas concentrations. Here, increasing climate change severity corresponds to increasing numbers, where SSP585 corresponds to fossil-fueled development while the SSP126 would require substantial mitigation efforts on a global level to achieve. The GFDL-ESM4 model provides temperatures for previous years for each of the four SSP scenarios, starting in 2015, (i.e., each scenario has different historical temperature data for those years). We use the SSP585 scenario for our historical temperatures (most closely corresponding to RCP (Representative Concentration Pathway) 8.5 from CMIP-5), because this trajectory is thought to most closely align with the carbon dioxide emissions from those years [32, 33].

**Fig 1:**
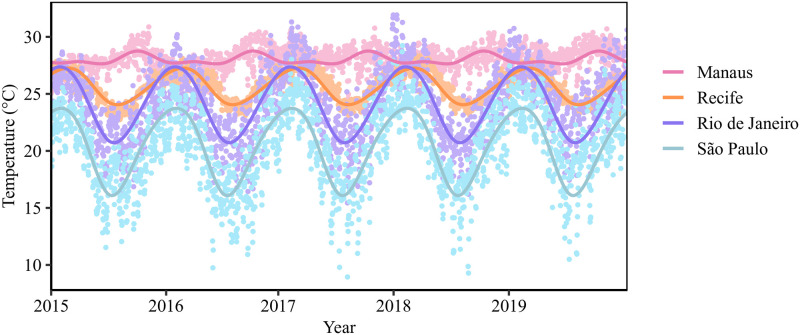
Daily temperature in 4 Brazilian cities, 2015–2019. Periodic cubic spline models are fit to the data for Manaus, Recife, Rio de Janeiro, and São Paulo to develop mean seasonal temperature models.

We summarized the historical and projected temperature data in two ways using period cubic B-splines. First, we obtain both a baseline seasonal temperature time series by taking the average temperature for each day across the five years of the model output (2015–2019; Fig 1), and a 2045–2049 projected year-round temperature dataset from the averages of the years 2045–2049 (Section 1, Fig A in S1 Appendix). By smoothing over the five years, we projected average climate and smooth any anomalies that occur in the projections for 2045–2049, giving an estimation of overall changes in risk by the second half of the 2040s as compared to the recent past. Second, to capture the year-to-year variation, we also fit the periodic splines to each of the five years separately for both the historical and projected temperatures to better understand reasonable likely deviation from the mean projection (Section 2, Fig B in S1 Appendix). The period cubic B-splines were fit to the temperature data using the pbs package [34] in R (v4.0; R Foundation for Statistical Computing; Vienna, Austria).

### Infectious disease transmission model

We modified an existing vector-borne infectious disease transmission model [22] to include birth and death processes. The basic model structure comprises an SLIR (susceptible, latent, infectious, recovered) model for human transmission and SLI model for mosquito transmission, using standard exponential birth and death processes for the human population. The model tracks the numbers of susceptible *S*_*h*_, latently infected *L*_*h*_, infectious *I*_*h*_, and recovered *R*_*h*_ humans (with total human population *N*_*h*_), as well as the number of susceptible *S*_*m*_, latently infected *L*_*m*_, and infectious *I*_*m*_ mosquitoes. Our model includes three temperature-independent, human parameters: the birth/death rate *μ*_*h*_, the transition rate from latency to infectiousness *σ*_*h*_ (which we assumed to be two days less than the intrinsic incubation period, as infectiousness precedes symptom onset [35]), and the recovery rate *γ*. The birth and death rates *μ*_*h*_ were fixed to single values based on current life expectancy for this analysis rather than projected; because the model is focused on epidemic potential (see below) rather than simulation, the results are not sensitive to these values (impacting the results only in the probability that a latent or infected individual may die before recovery).

Mathematical modeling allows us to estimate how the various temperature dependencies of vector traits combine to affect disease risk. To that end, our model includes eight temperature-dependent (*T*) *A. aegypti* mosquito parameters, five of which are independent of the pathogen: biting rate *a*(*T*), the number of eggs laid per day *ϵ*(*T*), the probability of egg to adult survival *θ*(*T*), the egg to adult development rate *ρ*(*T*), and the adult mosquito mortality rate *μ*_*m*_(*T*). One temperature dependent mosquito parameter not included in our eight parameters is the carrying capacity *K*(*T*), which is the maximum number of mosquitoes that the environment can sustain. This parameter can be modeled as a function of the other vector parameters [22] (see Section 3 in S1 Appendix) and therefore does not appear in the $R_{0}\left(T\right)$ formula we derive.

The three additional temperature-dependent parameters depend on the specific pathogen: the extrinsic incubation rate, that is the latency to infectiousness rate *σ*_*m*_(*T*), the per bite probability of pathogen transmission from mosquito to human *π*_*mh*_(*T*), and the per bite probability of pathogen transmission from human to mosquito *π*_*hm*_(*T*). We define vector competence as the product of *π*_*mh*_(*T*) and *π*_*hm*_(*T*), denoted (*π*_*hm*_*π*_*mh*_)(*T*). For Zika we have temperature-dependent estimates for the vector competence product but not the constituent parameters.

We used the thermal response curves fit by Mordecai et al. [16, 18], whose temperature-dependence are described by one of the four formulas: a Brière $\left(cT\left(T-T_{0}\right)\sqrt{\left(T_{m}-T\right)}\right)$, quadratic (*c*(*T* − *T*_*m*_)(*T* − *T*_0_)), inverse quadratic (*c*(*T* − *T*_*m*_)(*T* − *T*_0_))^−1^, or constant *c*, as appropriate for the shape of the relationship in the data (Table 1). *T*_0_ and *T*_*m*_ are the minimum and maximum temperatures for which a given parameter takes on a non-zero value. The parameter *c* is fit to the data [16, 17]. Plots of the temperature dependence of the biting rate *a*, the extrinsic incubation rate *σ*_*m*_, and the vector competence (*π*_*hm*_*π*_*mh*_) are given in Section 4, Fig C in S1 Appendix), distinguishing between dengue and Zika where appropriate.

**Table 1:** Parameters of the temperature-dependent vector-borne arbovirus disease transmission model. Adult mosquito mortality rate and extrinsic incubation period were refit by the authors (indicated by ^†^and given in Section 4, Fig D and E in S1 Appendix), while the remaining traits were taken directly from the source listed.

**Temperature-dependent parameters (Zika and dengue)**						
**Parameter**	**Definition**	**Source**	**Function**	*T*_0_	*T*_*m*_	*c*
*a*	biting rate (day^−1^)	[16]	Brière	13.35	40.08	2.02E-4
*ϵ*	eggs laid per female (day^−1^)	[16]	Brière	14.58	34.61	8.56E-3
*θ*	probability of mosquito egg to adult survival	[16]	Quadratic	13.56	38.29	-5.99E-3
*ρ*	mosquito egg to adult development rate (day^−1^)	[16]	Brière	11.36	39.17	7.86E-5
*μ*_*m*_	adult mosquito mortality rate	[16, 17]^†^	Inverse	8.53	38.07	-1.68E-1
**Temperature-dependent parameters (Zika)**						
$\sigma_{m}^{z}$	virus extrinsic incubation rate	[17]	Brière	18.27	42.31	1.74E-4
(*π*_*hm*_*π*_*mh*_)^*z*^	vector competence	[17]	Quadratic	22.72	38.38	-3.54E-3
**Temperature-dependent parameters (dengue)**						
$\sigma_{m}^{d}$	virus extrinsic incubation rate	[16]^†^	Brière	10.68	43.09	6.91E-5
$\pi_{mh}^{d}$	probability of transmission to human (per bite)	[16]	Brière	17.05	35.83	8.49E-4
$\pi_{hm}^{d}$	probability of transmission to vector (per bite)	[16]	Brière	12.22	37.46	4.91E-4
**Temperature-independent parameters (Zika and dengue)**						
*μ*_*h*_	human birth/death rate (day^−1^)	[37]	Constant	—	—	(75.7 × 365)^−1^
$\frac{N_{m}}{N_{h}}$	Ratio of *A. aegypti* to humans	[38]	Constant	—	—	9.75E-1
**Temperature-independent parameters (Zika)**						
$\sigma_{h}^{z}$	human latency rate (*day*^−1^)	[35, 39]	Constant	—	—	1/4
*γ* ^*z*^	human recovery rate (*day*^−1^)	[35, 39]	Constant	—	—	1/5
**Temperature-independent parameters (dengue)**						
$\sigma_{h}^{d}$	human latency rate (*day*^−1^)	[35]	Constant	—	—	1/4
*γ* ^*d*^	human recovery rate (*day*^−1^)	[35]	Constant	—	—	1/5

We refit two of the temperature-dependent parameters: the extrinsic incubation period and mosquito lifespan. Because the mosquito mortality rate should largely be independent of the pathogen, we merge the data from [16] and [36] to generate a temperature-dependent mosquito mortality *μ*_*m*_(*T*). Maximum likelihood estimates for the parameters *c*, *T*_0_, and *T*_*m*_ were obtained assuming mosquito lifetimes were Poisson distributed (Section 4, Fig D in S1 Appendix). Similarly, the extrinsic incubation rate was refit to exclude sources from papers which studied other arboviruses such as Yellow Fever. We parameterize the number of mosquitoes (*N*_*m*_) and the number of humans (*N*_*h*_) as a single parameter, $\frac{N_{m}}{N_{h}}$, corresponding to the density of mosquitoes (i.e., the number of mosquitoes per human).

The parameters are summarized in Table 1, and the model equations are given below.

$$\begin{array}{c} \begin{array}{c} \frac{dS_{h}}{dt}=\mu_{h}\cdot N_{h}-a\left(T\right)\cdot \pi_{mh}\left(T\right)\cdot \frac{I_{m}}{N_{h}}\cdot S_{h}-\mu_{h}\cdot S_{h}\\ \frac{dL_{h}}{dt}=a\left(T\right)\cdot \pi_{mh}\left(T\right)\cdot \frac{I_{m}}{N_{h}}\cdot S_{h}-\sigma_{h}\cdot L_{h}-\mu_{h}\cdot L_{h}\\ \frac{dI_{h}}{dt}=\sigma_{h}\cdot L_{h}-\gamma \cdot I_{h}-\mu_{h}\cdot I_{h}\\ \frac{dR_{h}}{dt}=\gamma \cdot I_{h}-\mu_{h}\cdot R_{h}\\ \frac{dS_{m}}{dt}=\epsilon \left(T\right)\cdot \theta \left(T\right)\cdot \rho \left(T\right)\cdot \mu_{m}{\left(T\right)}^{-1}N_{m}(1-\frac{N_{m}}{K(T)})-\\ (a\left(T\right)\cdot \pi_{hm}\left(T\right)\cdot \frac{I_{h}}{N_{h}}+\mu_{m}\left(T\right))\cdot S_{m}\\ \frac{dL_{m}}{dt}=a\left(T\right)\cdot \pi_{hm}\left(T\right)\cdot \frac{I_{h}}{N_{h}}\cdot S_{m}-\left(\sigma_{m}\left(T\right)+\mu_{m}\left(T\right)\right)L_{m}\\ \frac{dI_{m}}{dt}=\sigma_{m}\left(T\right)\cdot L_{m}-\mu_{m}\left(T\right)\cdot I_{m} \end{array} \end{array}$$ (1)

### Basic reproduction number

The basic reproduction number $R_{0}$ is a measure of the epidemic potential of an infectious disease system [40, 41]. It represents the expected number of secondary infections caused by a single infectious case over their infectious period in an otherwise susceptible population. If $R_{0}>1$, an epidemic is expected to grow and if $R_{0}<1$, an epidemic is expected to die out. $R_{0}$ is an appropriate metric for our projections because there is too much uncertainty on what specific circulation patterns will be over time and in population-level immunity to project specific outbreak dynamics in 30 years. Our approach instead focuses on transmission potential. Even if there is substantial population immunity suppressing Zika and dengue circulation, understanding transmission potential is still useful and can inform arboviral disease potential more broadly.

In the context of vector-borne disease systems, there is some subtlety to the interpretation of $R_{0}$: strictly speaking, a disease generation-based $R_{0}$, as derived by the next generation matrix (NGM) [42, 43] and denoted below as $R_{0}^{NGM}$, treats hosts (humans) and vectors (mosquitoes) as equally important, essentially taking the mean of human-to-vector infections and vector-to-human infections. Because we observe human cases, only, it is usually preferable and more interpretable to use the expected number of new human infections per infectious human, namely $R_{0}={\left(R_{0}^{NGM}\right)}^{2}$. This formulation is consistent with classic approaches [44]. We use the next generation method to derive a formula for $R_{0}^{NGM}$ and thus this latter temperature-dependent $R_{0}\left(T\right)$ for our model (see Section 5 in S1 Appendix).

$$\begin{array}{c} R_{0}\left(T\right)={\left(R_{0}^{NGM}\left(T\right)\right)}^{2}=\frac{a\left(T\right)\cdot \pi_{mh}\left(T\right)\cdot \sigma_{m}\left(T\right)}{\mu_{m}\left(T\right)\left(\sigma_{m}\left(T\right)+\mu_{m}\left(T\right)\right)}\cdot \frac{a\left(T\right)\cdot \pi_{hm}\left(T\right)\cdot \sigma_{h}}{\left(\sigma_{h}+\mu_{h}\right)\left(\gamma +\mu_{h}\right)}\cdot \frac{N_{m}}{N_{h}} \end{array}$$ (2)

Incorporating the virus-specific parameter values into this expression, we derive values for Zika,

$$\begin{array}{c} R_{0,z}\left(T\right)=\frac{{\left(a\left(T\right)\right)}^{2}\cdot {\left(\pi_{hm}\pi_{mh}\right)}^{z}\left(T\right)\cdot \sigma_{m}^{z}\left(T\right)\cdot \sigma_{h}^{z}}{\mu_{m}\left(T\right)\left(\sigma_{m}^{z}\left(T\right)+\mu_{m}\left(T\right)\right)\left(\sigma_{h}^{z}+\mu_{h}\right)\left(\gamma^{z}+\mu_{h}\right)}\cdot \frac{N_{m}}{N_{h}}, \end{array}$$ (3)

and dengue,

$$\begin{array}{c} R_{0,d}\left(T\right)=\frac{{\left(a\left(T\right)\right)}^{2}\cdot \pi_{mh}^{d}\left(T\right)\cdot \pi_{hm}^{d}\left(T\right)\cdot \sigma_{m}^{d}\left(T\right)\cdot \sigma_{h}^{d}}{\mu_{m}\left(T\right)\left(\sigma_{m}^{d}\left(T\right)+\mu_{m}\left(T\right)\right)\left(\sigma_{h}^{d}+\mu_{h}\right)\left(\gamma^{d}+\mu_{h}\right)}\cdot \frac{N_{m}}{N_{h}}, \end{array}$$ (4)

as a function of the pathogen-specific, temperature-dependent parameters.

To assess how each parameter influences the $R_{0}\left(T\right)$ curves for Zika and dengue and the city-specific projections, we also conducted sensitivity analysis. For a detailed description of the sensitivity analysis, see Sections 6 and 7 in S1 Appendix. Briefly, we investigated the impact of removing the temperature-dependence of each parameter (i.e., fixing it to its mean value over its non-zero values) on $R_{0}\left(T\right)$ and on the results for the city-specific projections.

## Results

### Temperature-dependent basic reproduction numbers

**Fig 2:**
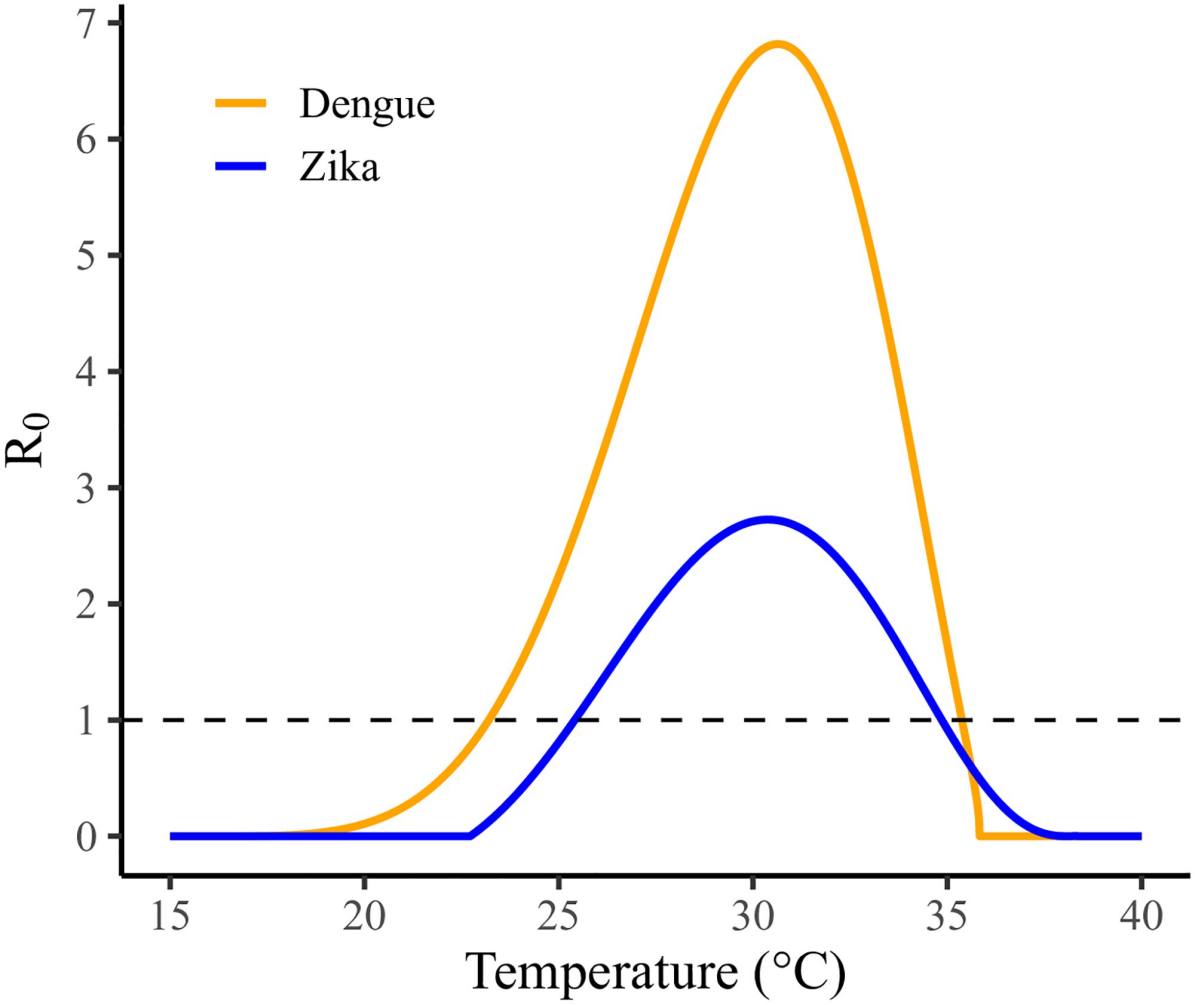
Temperature-dependent $R_{0}\left(T\right)$ for Zika and dengue.

The temperature-dependent shape of the $R_{0}\left(T\right)$ curve is similar for Zika and dengue (Fig 2). For example, the peak $R_{0}$ occurs at approximately 30.5°*C*. On the other hand, $R_{0}$ increases above 1 at a cooler temperature for dengue compared to Zika (23°*C* vs 25°*C* respectively), and the peak $R_{0}$ value is greater for dengue than Zika (6.8 vs 2.7). In our sensitivity analysis, we find that mosquito biting rate is the largest driver of the magnitude of $R_{0}$ (Section 6, Fig F in S1 Appendix) and mosquito lifespan has the largest influence on the temperature at which it reaches its peak value (Section 6, Fig Gb and d in S1 Appendix), whereas vector competence (the probability of transmission to human times the probability of transmission to vector) more greatly drives the steepness (i.e., sensitivity to small changes in temperature) of the $R_{0}$ curve (Section 6, Fig Ga and c in S1 Appendix). The extrinsic incubation period has little impact on the temperature-dependent $R_{0}\left(T\right)$ for each virus. Note that the $R_{0}$ metric considers a fully susceptible population, and the effective reproduction numbers for real populations decrease proportionally to the fraction of the population that is immune.

### Risk projections based on 5-year temperature data

**Fig 3:**
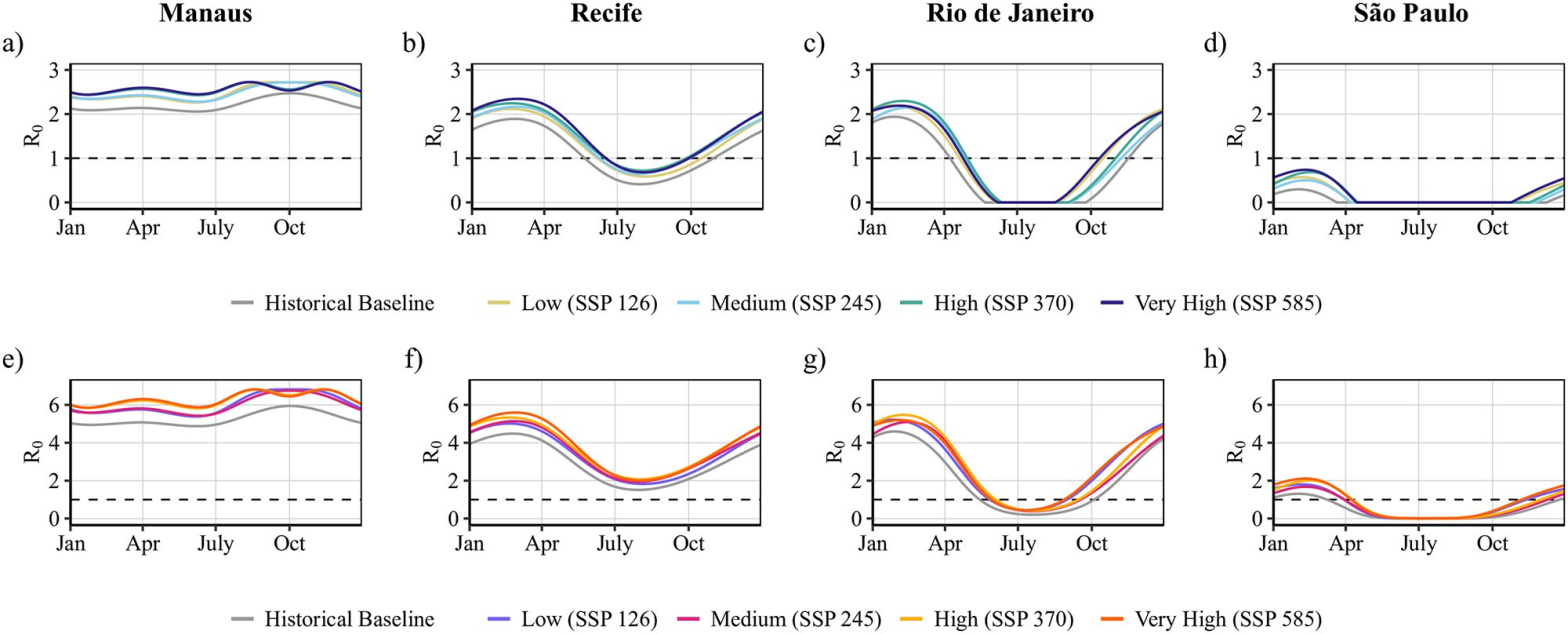
Projection of seasonal epidemic potential $R_{0}\left(T\left(t\right)\right)$ by 2045–2049. Projections are given for Zika (a-d) and dengue (e-h) for each city and climate change scenario.

The climate change scenarios project a year-round increase of $R_{0}$ by 2045–2049, with varying degrees of difference among the risk projections between the specific SSP scenarios (Fig 3). Exceptions include the extreme temperatures seen in the warm season (i.e., September through November) in Manaus and the cool season (i.e., June through September) in Rio de Janeiro and São Paulo. For Manaus, we predict the annual Zika $R_{0}$ range, currently 2.1–2.5, to shift to 2.3–2.7, for Recife we project the range to shift from 0.4–1.9 to 0.6–2.3, for Rio de Janeiro to shift from 0–1.9 to 0–2.3, and for São Paulo to shift from 0–0.3 to 0–0.7. The increase in $R_{0}$ is not uniform throughout the year as can be seen in the graphs for Rio de Janeiro in particular (Fig 3c and 3g), where the $R_{0}$ value increases by a far larger amount during the months of October through April than it does earlier in the year ($R_{0}$ remains 0 throughout the winter months in all scenarios, but increases as high as 0.8 in the spring and summer months). To a lesser extent, $R_{0}$ increases are also non-uniform for Recife (increasing around 0.1 earlier in the year and as high as 0.5 by late winter, (Fig 3b and 3f)). These effects are due to a combination of the non-uniform temperature changes in the temperature projection data over the year and the non- linearity of the $R_{0}$ formula. We see some minor attenuation of risk because of higher temperatures across the risk projections in the warmest months in Manaus (Fig 3a and 3e), where temperatures are projected to reach just above 35°C in the SSP585 scenario. However, in this scenario, the peak risk still far surpasses that of the baseline risk, occurring at two different times in the year corresponding to the bookends of the observed dip in risk (around September and November).

Our baseline risk estimates for Rio de Janeiro and Recife suggest that the current risk season for Zika, i.e., the time for which $R_{0}>1$, is late spring through fall (i.e., December through March) which is largely consistent with data from the 2015–16 outbreak [45, 46]. Dengue follows a similar trend, but with a longer risk season. Our risk projections suggest that the arbovirus risk season for Rio de Janeiro will increase by approximately 2–3 months by 2045–2049 and that the Zika risk seasons in Recife will increase by around 2 months. In São Paulo, the $R_{0}$ for dengue more reliably sits above 1 during the beginning of the year in our projections for 2045–2049, peaking at 2.1 in SSP585, nearly double the peak $R_{0}$ value from the baseline.

Our city-specific sensitivity analysis (Section 7, Fig H and I in S1 Appendix,) suggests that the temperature-dependent parameters have varying degrees of impact on our $R_{0}$ projections for each city. For example, holding vector competence, biting rate, and mosquito lifespan constant has the largest absolute impact on the $R_{0}$ in the warmer cities (especially Manaus), while São Paulo’s results are less impacted by the sensitivity analysis. Like the sensitivity analysis for $R_{0}\left(T\right)$, we see that the extrinsic incubation period has little impact on any of the results.

### Risk projections based on individual-year data

**Fig 4:**
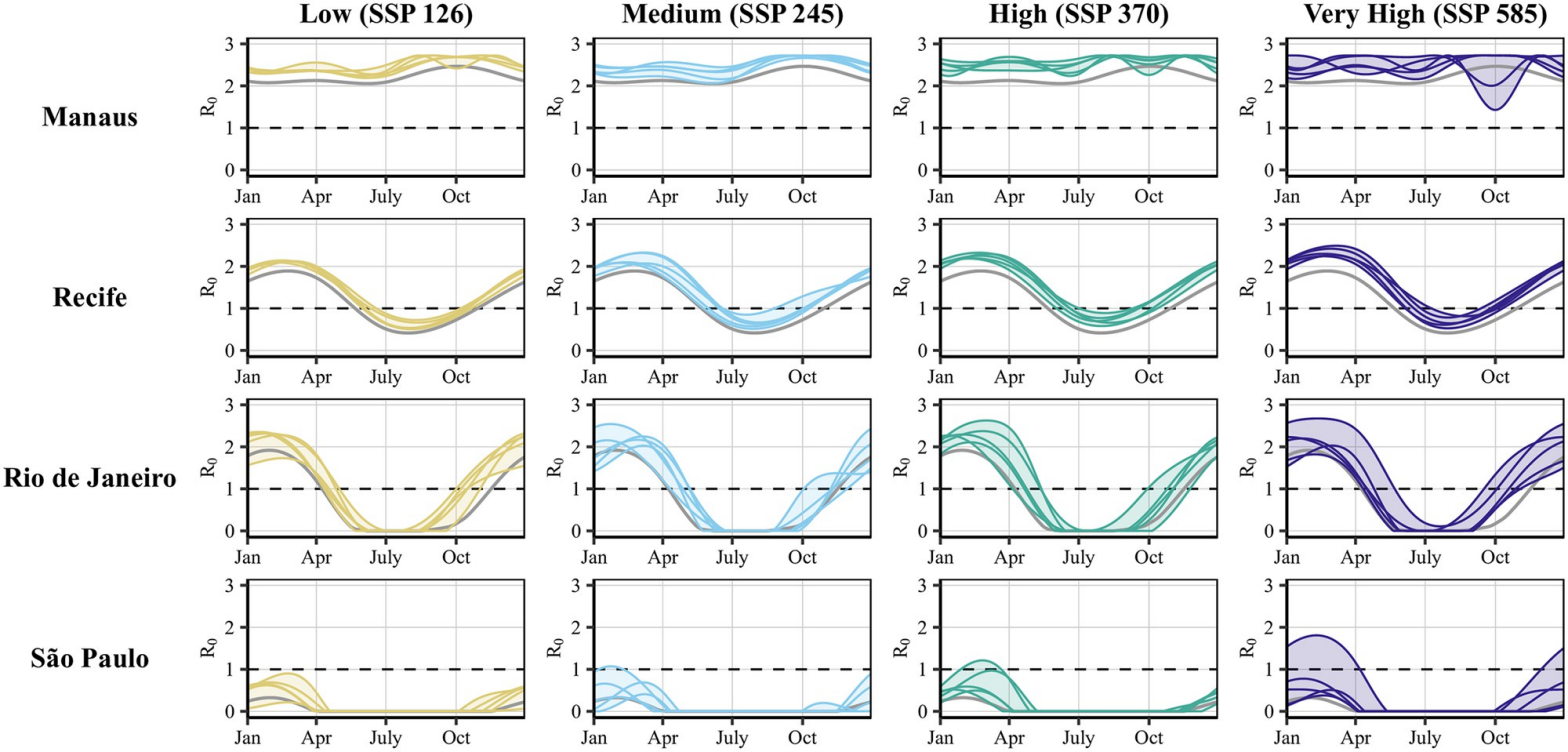
Year-to-year heterogeneity in projected Zika risk across climate change scenarios. Each panel shows projections of the seasonal temperature in years 2015–-2019 for a specific climate change scenario and each city, demonstrating year-to-year heterogeneity in projected risk. Grey lines corresponding to the historical baseline are plotted for comparison. Ribbons were added to highlight the vertical spread in the lines for each year.

Our risk projections based on individual year data (Fig 4) highlight the heterogeneity of the Zika risk projections from year-to-year (the corresponding figure for dengue can be found in Section 8, Fig J in S1 Appendix). The projections for Manaus contrast with the projections from the other three cities, which still show largely consistent increase in disease risk throughout the year for each year. For example, Manaus sees a sharp decrease in risk in the spring (September through November) for two years in the SSP585 scenario, demonstrating potentially erratic shifts in peak risk seasons for this city to earlier in the year.

São Paulo’s risk is also highly variable between the different years and SSP scenarios, highlighting important distinctions between each of the scenarios. For example, our projections show a dramatic difference in one of the years in the SSP585 scenario compared to the other scenarios. These results suggest that the year-to-year heterogeneity in temperature and thus on arbovirus disease risk in the future will likely depend on regional climate factors and year-specific weather patterns.

## Discussion

In this work, we use a temperature-dependent transmission model to investigate the impacts of climate change on Zika risk in Brazil, highlighting both geographic and year-to-year variation in projected risk. Zika and dengue’s temperature profile for $R_{0}$ peaks at a relatively high temperature, around 30°*C*; therefore, prior work has suggested that climate change will both increase their transmission potential and geographic extent of transmission [20]. When examining the impact of climate change projections on Brazil, we find general agreement with this expectation but also find variability across different climatic regions within the country. This variability across different climate zones is evident in Fig 4, which shows that Manaus is a region on the cusp of experiencing a decrease in arbovirus risk at certain times of the year in certain years, while both Recife and Rio de Janeiro show large increases in risk throughout the year. In places like Recife and Rio de Janeiro, we project the extension of the risk season. In São Paulo, a city that lies on the borderline of reliable *A. aegypti* suitability [3, 47, 48], we see that it is likely that future arboviral risk will depend on how the climate changes. These results highlight that transmission is likely to expand into geographic regions with cooler climates. Regions with current temperatures that are too cold to sustain year-round transmission will become increasingly vulnerable to newly seeded outbreaks sparking seasonal epidemics.

Temperature-dependent $R_{0}\left(T\right)$ curves, used here and in other studies, indicate that there is a potential for increasing temperatures to have a protective effect. The curves for dengue and Zika begin to decrease sharply after they peak at around 30°C, both decreasing to 1 by around 35°C (95°F). Of the four cities in the analysis, Manaus is the only city to reach this peak temperature, and even in the high emission scenario, the maximum temperature in our projections is only briefly above 35°*C* at the beginning of October. Thus, even in regions with warmer tropical rain forest climates like Manaus, our results show that in most regions climate change is not likely to have a substantial or consistent protective effect on arbovirus transmission.

Over the past decades, numerous studies have looked at the impact of temperature changes on vector-borne disease transmission [3, 5, 13, 21, 49–52]. There is general consensus among these studies that both dengue and Zika will spread into areas that are becoming increasingly suitable for transmission (e.g., the Southeastern United States) and that risk will increase in currently endemic areas. The temperature-dependence of mosquito-borne disease transmission is a complicated mix of multiple processes, each of which generally has a non-linear relationship with temperature. Several studies concur that 26–29°C is the optimal temperature window for arbovirus transmission [16, 17, 53, 54]. Zika and dengue lie on the higher end of this range [21], at around 30°*C*, which is consistent with our estimates. Our $R_{0}\left(T\right)$ estimates also span ranges that are consistent with empirical estimates: a systematic literature review on the basic reproduction numbers for dengue and Zika found the $R_{0}$ of Zika (mean 3.0) to be lower than the $R_{0}$ of dengue (mean 4.3) in tropical climates, and our estimated values are well within the substantial variation in individual study estimates [55]. Our $R_{0}$ for Zika is just below 3.0 even at its highest value.

The variability between the various SSP scenarios seen in Fig 4 along with steepness of the temperature-dependent $R_{0}$ curves (Fig 2) underscore the severe consequences of small deviations in temperature projections with regards to arbovirus risk. That is, our ability to control our emissions to prevent even small temperature increases could have massive benefits relating to mosquito-borne illnesses. That being said, by 2045–2049, even the best-case scenario (SSP126) corresponds to both lengthening of the risk season—particularly for Rio de Janeiro—and increases in overall disease pressure, indicating that international climate protection policy must be accompanied with national-level preparedness including increased surveillance, and diagnostic and treatment capacity.

For this analysis, we chose to focus on epidemic potential through the basic reproduction number. Previous work has demonstrated that beyond epidemic potential, there will likely be differences in epidemic dynamics, such as epidemic length, peak size, and final size [22]. However, for the long-term projections we provide here, $R_{0}$ is appropriate—there are too many unknowns in terms of what population-level immunity will look like to make reasonable projections of specific dynamics. Indeed, it is unclear whether these arboviruses will be circulating in the coming decades and whether new pathogens will emerge. Thus, one strength of this study is the side-by-side comparison of dengue and Zika risk, which gives a broader look at arbovirus epidemic potential, regardless of the specific pathogen. Another strength of our work is the generalizability of our modeling approach to other geographic areas; the temperature-dependent $R_{0}\left(T\right)$ we derived here could be implemented for other geographic regions of interest by leveraging the appropriate temperature data. However, we note that several model parameters would be improved with data specific to the geographic region. For example, several thermal-dependent traits for *Ae. aegypti* including lifespan and reproductive rate are known to vary between regions [56], but we did not have information on strains specific to Brazil for this study. Future work on temperature-dependent traits for region-specific mosquito strains would greatly improve the generalizability of our approach for other geographic regions. Finally, our study also uses a fine temporal granularity, which gives us the ability to provide a more in depth understanding of year-round dynamics and investigate arbovirus risk as a dynamic value that changes over the course of a month or year.

However, our work is limited by its sole focus on temperature. Climate change is likely to impact humidity and rainfall, and population density will also likely change in the future. We do not account for these factors in our projections. Due to its additional impact on human behavior (e.g., water storage practices in response to drought or flooding), interaction with the physical environment (e.g., water collecting in puddles or trash provides larval habitat), and large uncertainty in projections relative to temperature [57, 58], precipitation is a more complicated climatic factor to include in models than temperature and including this would have introduced tremendous uncertainty to the results, even using state-of-the-art model projections. Another limitation is our inability to predict future population birth rates and changes in the mosquito-to-human population density ratio, which may impact the epidemic potential of Zika and other arboviruses. There is also uncertainty in the exact temperature relationship for many parameters, including the mosquito biting rate, which is particularly difficult to estimate empirically. Moreover, in many cases, temperature-dependent traits were estimated in lab conditions and may differ significantly from real-world settings, because of larval resource availability, diurnal temperature fluctuations, and other real-world factors that are known to impact vector traits [59–61].

## Conclusion

Climate forecasts coupled with transmission models, as used in this study, provide a source of evidence to guide future planning to mitigate health impacts due to climate change. Local and national health departments can leverage these sources in preparing for increases in transmission pressure due to climatic warming. Our work contributes to the larger literature of climate change health impacts by exploring the likely heterogeneities in these health impacts both across climatic regions within a country and from year-to-year. Greater flexibility and adaptability of arbovirus response and prevention may be necessary to accommodate spatial and temporal heterogeneity in risk projections, especially in a country with as much climatic diversity as Brazil.

## Supporting information

S1 Appendix Supporting information. In the supporting information, we provide the periodic spline fits to the individual years in each city, provide the periodic spline fits to the five-year temperatures under each of the climate change scenarios in each city, discuss the temperature-dependent mosquito carrying capacity, give the temperature-dependent parameter models as well as the fits to the data were applicable, and provide the individual-year risk projections for dengue (analogous to Fig 4).
(PDF)
