# Supplementary material for: Long-term projections of the impacts of warming temperatures on Zika and dengue risk in four Brazilian cities using a temperature-dependent basic reproduction number
Source: PLoS Negl Trop Dis. 2023 Apr 27;17(4):e0010839. doi: 10.1371/journal.pntd.0010839 (PMC10138270; doi:10.1371/journal.pntd.0010839)
Supplement: S1 Appendix — In the supporting information, we provide the periodic spline fits to the individual years in each city, provide the periodic spline fits to the five-year temperatures under each of the climate change scenarios in each city, discuss the temperature-dependent mosquito carrying capacity, give the temperature-dependent parameter models as well as the fits to the data were applicable, and provide the individual-year risk projections for dengue (analogous to Fig 4). (PDF) [file pntd.0010839.s001.pdf]

S1 Appendix for *Long-term projections of the impacts of warming temperatures on Zika and dengue risk in four Brazilian cities using a temperature-dependent basic reproduction number*

1 Climate scenarios

In Fig A, we plot periodic splines fit to forecasted temperatures based on the 5-year historical data and each each climate change emission scenario for each city. The projected temperatures are similar among the different climate change scenarios.

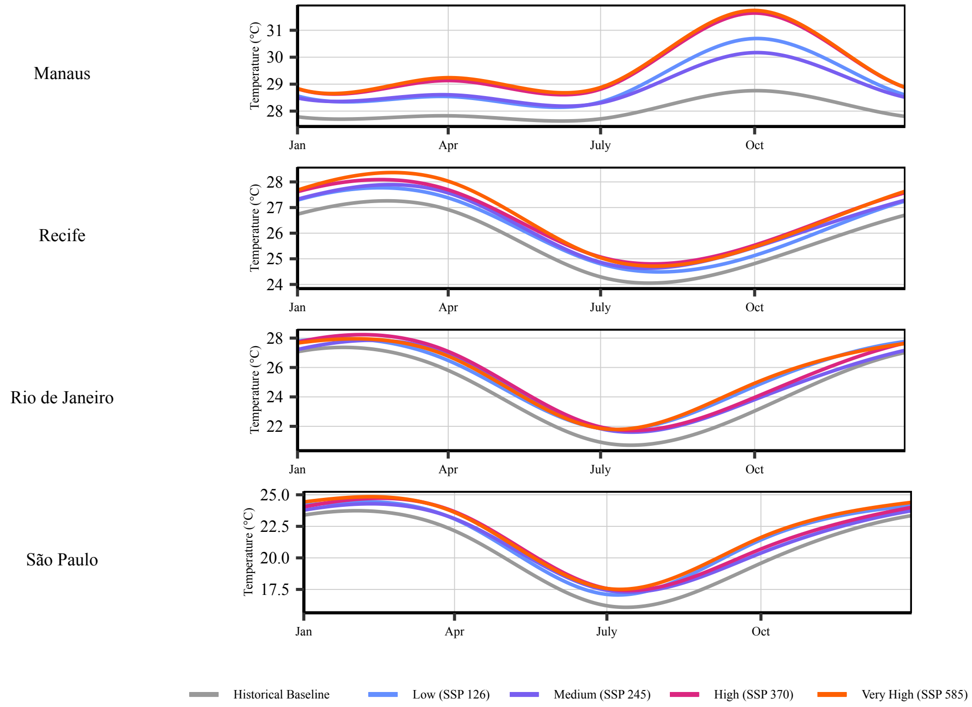

Figure A: **Projected seasonal temperatures under different climate change scenarios in each of the three Brazilian cities.**

2 Historical temperatures by year

In Fig B, we plot the best-fit periodic cubic spline approximations for the three Brazilian cities based on each individual year separately. The most notable yearly variation is the maximum temperature reached in Manaus.

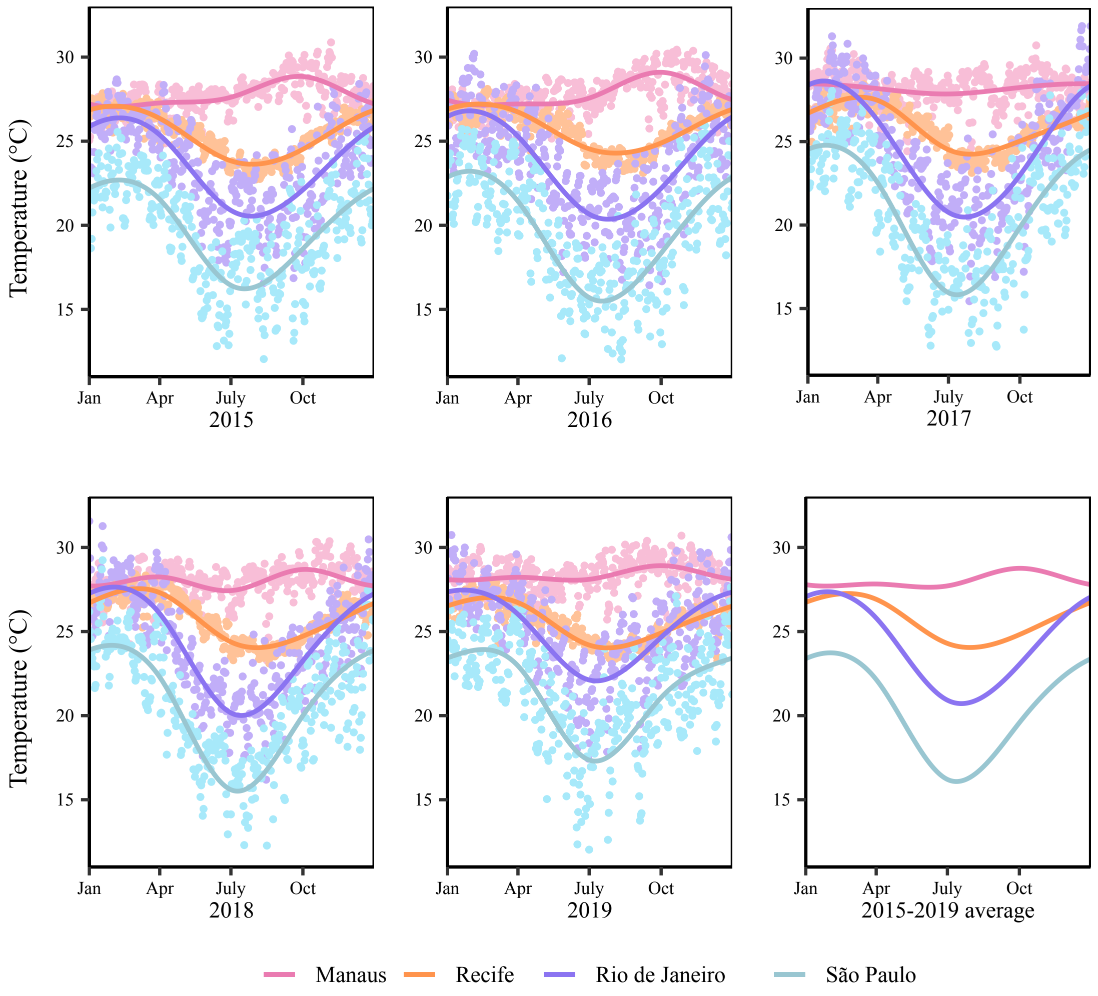

Figure B: **Yearly temperature and periodic splines, 2015–2019, for each of three Brazilian cities.**

3 Temperature dependent carrying capacity

The temperature dependent mosquito carrying capacity is defined as a function of other model parameters [1].

$$K(T) = \frac{\epsilon(T^*) \cdot \theta(T^*) \cdot \rho(T^*) \cdot \mu_m(T^*)^{-1} - \mu_m(T^*)}{\epsilon(T^*) \cdot \theta(T^*) \cdot \rho(T^*) \cdot \mu_m(T^*)^{-1}} \cdot N_m \cdot e^{\frac{E_a \cdot (T-T^*)^2}{\kappa_B \cdot (T+273) \cdot (T^*+273)}}, \tag{S1}$$

where T^* =29 °C, κ_B is the Boltzmann constant (8.617×10^{-5} eV/K), and E_A is the activation energy (set to 0.5) [1]. This parameter does not appear in the $\mathcal{R}_0$ formula, but would be relevant to model simulation.

4 Temperature dependent model parameters

We plot the temperature dependent model parameters. In Fig C, we plot the temperature dependent biting rate, extrinsic incubation rate, and vector competence by pathogen. In Fig D, we merge two previous studies to arrive at an estimated temperature dependent mosquito lifetime. Finally, in Fig E, we revisit the temperature dependent extrinsic incubation rate for dengue, focusing on studies solely of dengue.

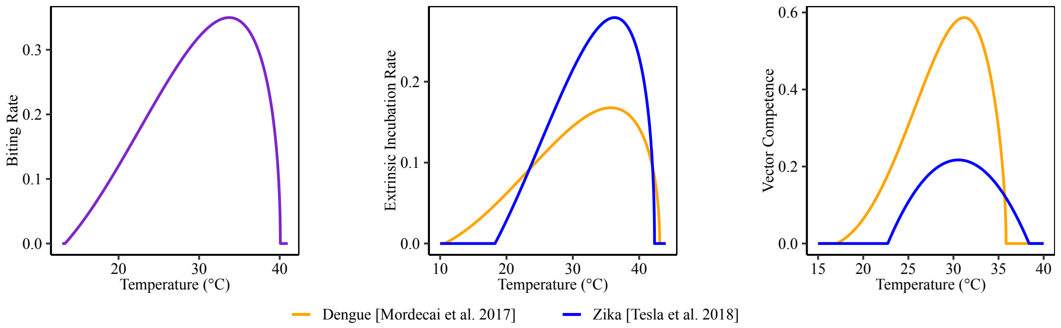

Figure C: **Temperature-dependent parameter values of the biting rate, extrinsic incubation rate, and the vector competence** [2, 3].

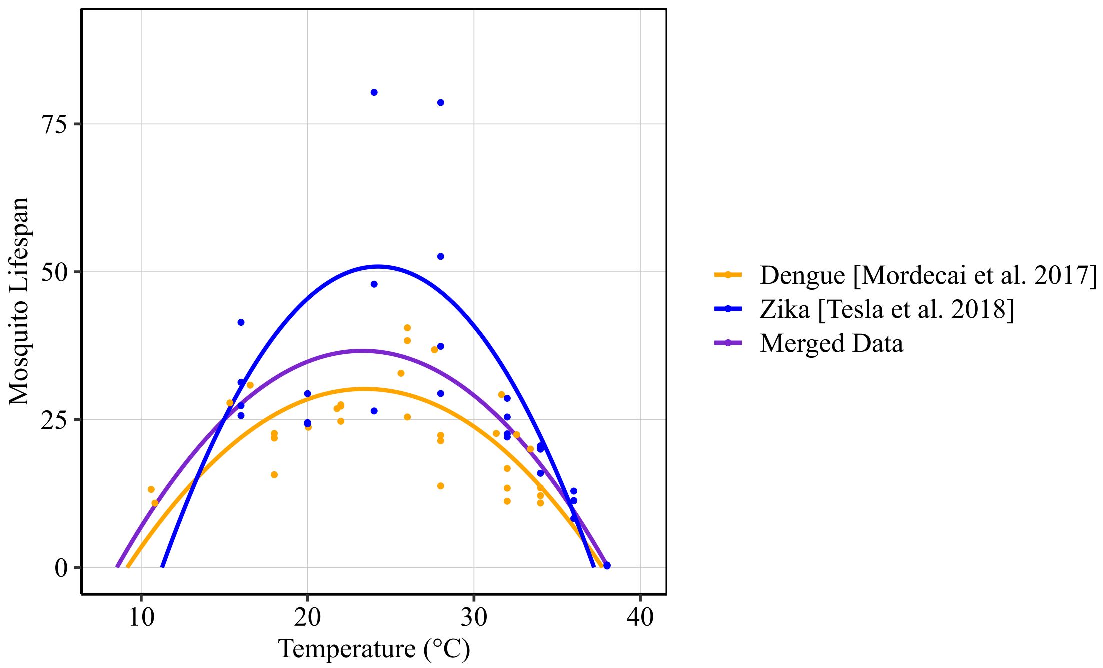

Figure D: **Estimates of temperature-dependent adult mosquito lifespan. Data were merged then fit using an inverse quadratic function.** The inverse quadratic function was parameterized using a Poisson maximum likelihood estimate fit to empirical lifespan data [2, 3].

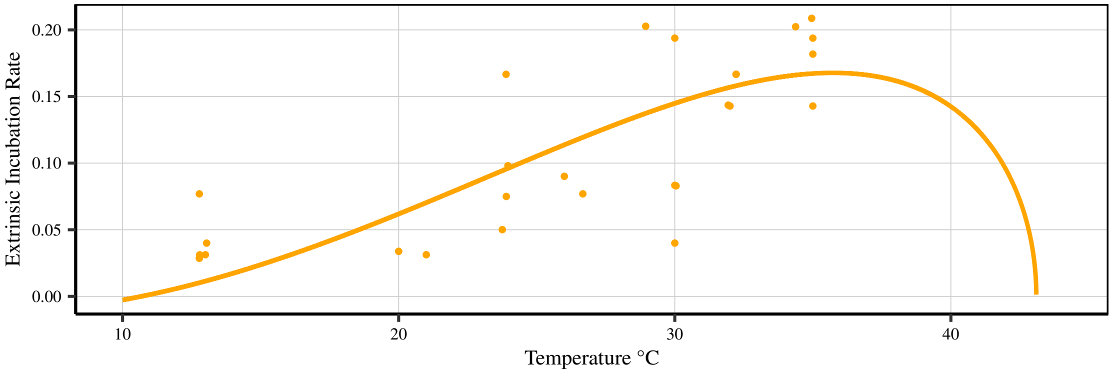

Figure E: **Estimated temperature dependent extrinsic incubation rate for dengue.** Temperature-dependent extrinsic incubation period data [2] were fit using a BriÃšre function parameterized by a Poisson maximum likelihood estimate.

5 Sensitivity analysis of $\mathcal{R}_0(T)$

First, we plot the $\mathcal{R}_0(T)$ curves holding each combination of the four temperature-dependent parameters constant at its mean value (giving 16 possible combinations). For each of the four parameters, we plot the 8 curves where it was held constant and the 8 where it follows the temperature-dependent distribution given in Table 1 (Fig F). Next, we assessed the width of the interval where $\mathcal{R}_0(T)$ is greater than half of its maximum value (full width at half maximum) for each curve and plot this for each parameter (Fig G). We do the same for the temperature at which the $\mathcal{R}_0(T)$ for each parameter combination reaches its peak value (Fig G).

In Fig F, we see that EIP (extrinsic incubation period) has the smallest impact on both the magnitude and the shape of $\mathcal{R}_0(T(t))$, while mosquito biting rate has a large impact on the magnitude. Fig G demonstrates that both EIP and lifespan have a small impact on the width of the $\mathcal{R}_0(T(t))$ distribution, while vector competence and biting rate have a lare impact. However, mosquito lifespan has the largest impact on which temperature the distribution reaches its peak $\mathcal{R}_0(T(t))$.

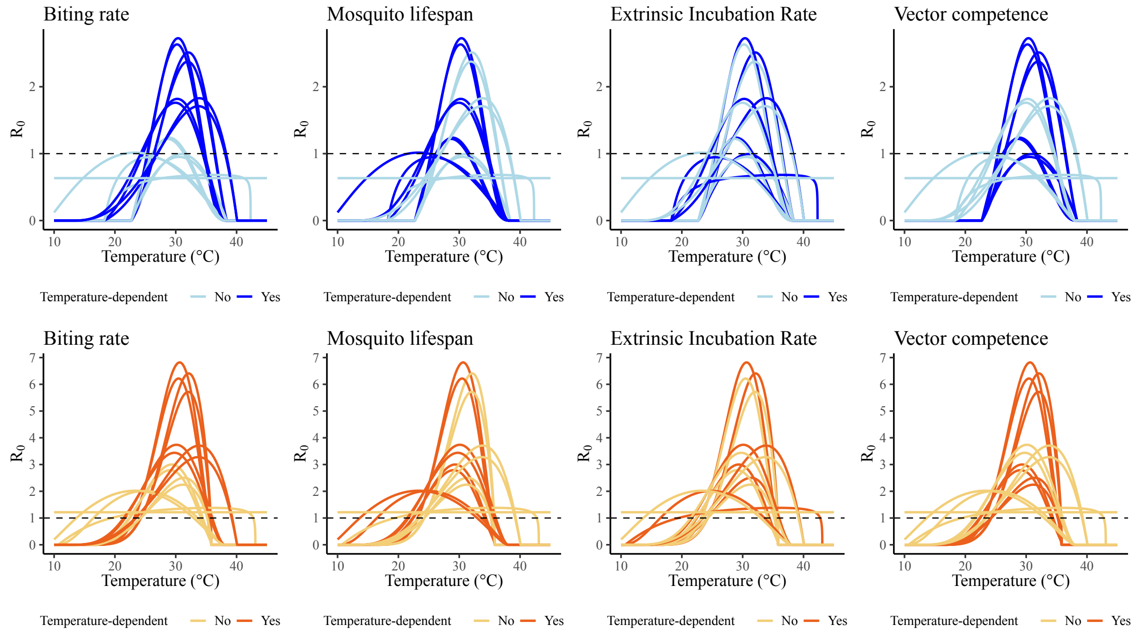

Figure F: **Temperature-dependent $\mathcal{R}_0(T(t))$ for Zika (top row) and dengue (bottom row) holding each combination of the 4 temperature-dependent parameters constant. The graph for each vector trait has two line colors, with the lighter color corresponding to the trait being help constant at its mean value.**

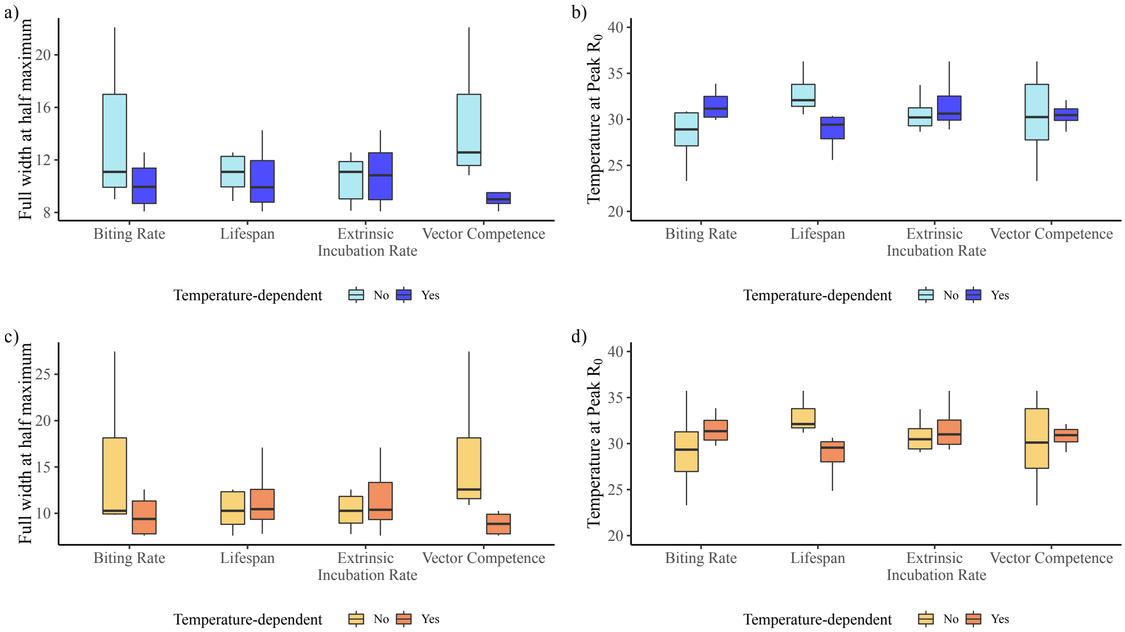

Figure G: **Impact of fixing each temperature-dependent parameter on the full width at half maximum ((a) and (c)) and the temperature at peak $\mathcal{R}_0(T(t))$((b) and (d)). The graph for each vector trait has two box plots, with the lighter color corresponding to the trait being help constant at its mean value.**

6 Sensitivity analysis of city-specific scenarios

Next, we show the sensitivity of the city-specific projections to the temperature-dependent parameters. Here, we remove the temperature-dependence of only one parameter at a time, corresponding to the parameter at the head of each column of Fig H (Zika) and Fig I (dengue). For each parameter, we show its impact on both the historical baseline and the highest SSP scenario (i.e., SSP 585).

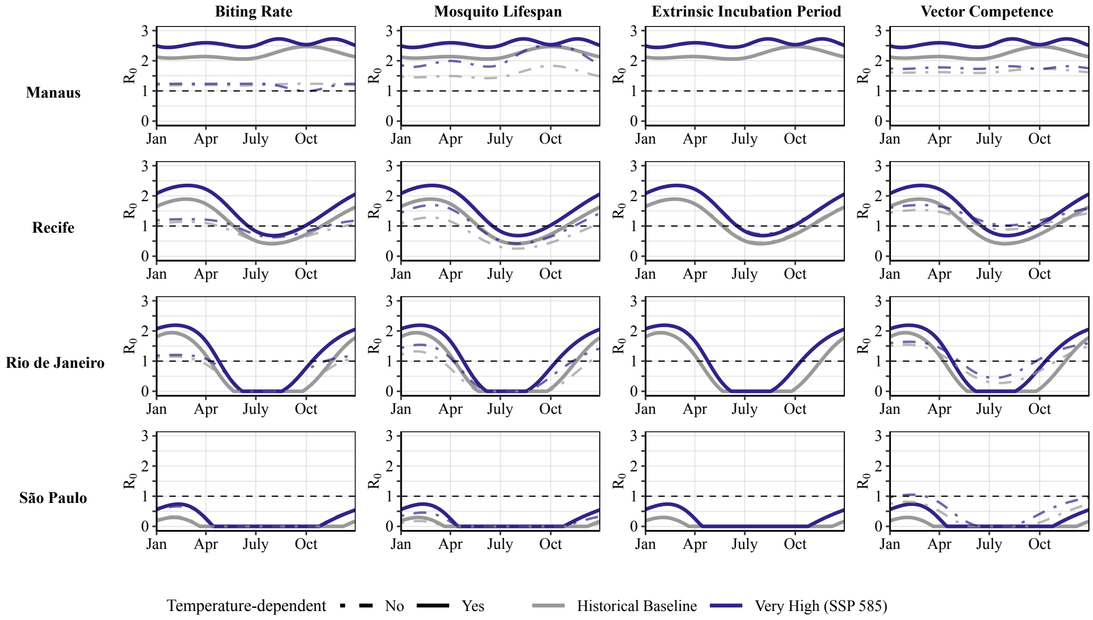

Figure H: **City-specific sensitivity analysis for Zika in each of the 4 cities.**

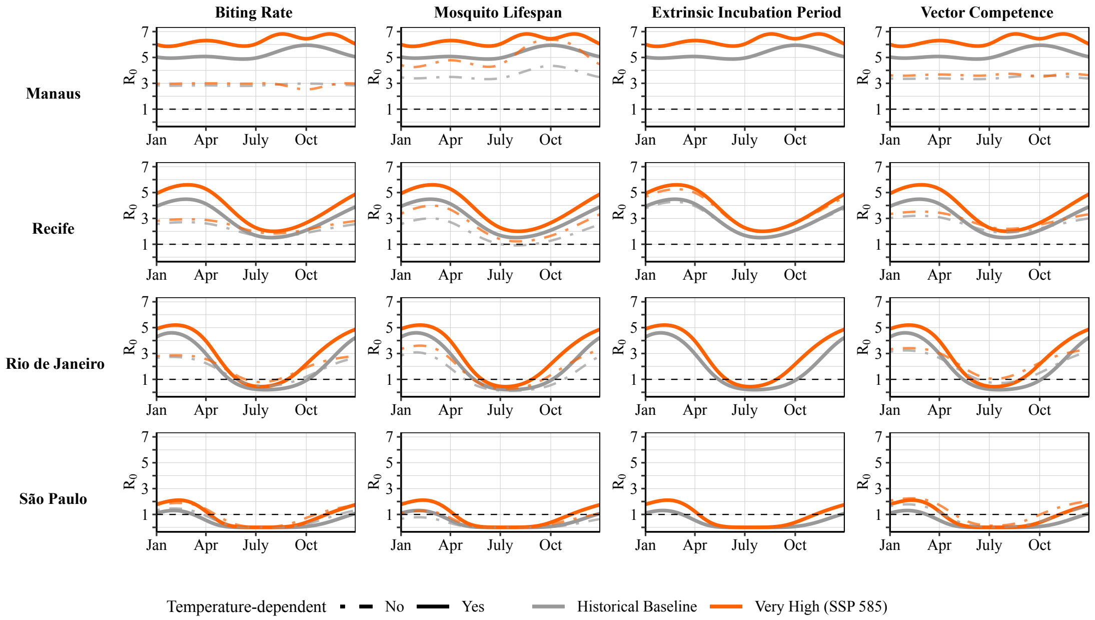

Figure I: **City-specific sensitivity analysis for dengue in each of the 4 cities.**

7 Risk projections based on individual-year data for dengue

In Fig J, we give the risk projections based on individual year data for dengue (analogous to Fig 4 in the main text for Zika). As with Zika, dengue risk in Manaus may be slightly depressed in the hottest month of some years.

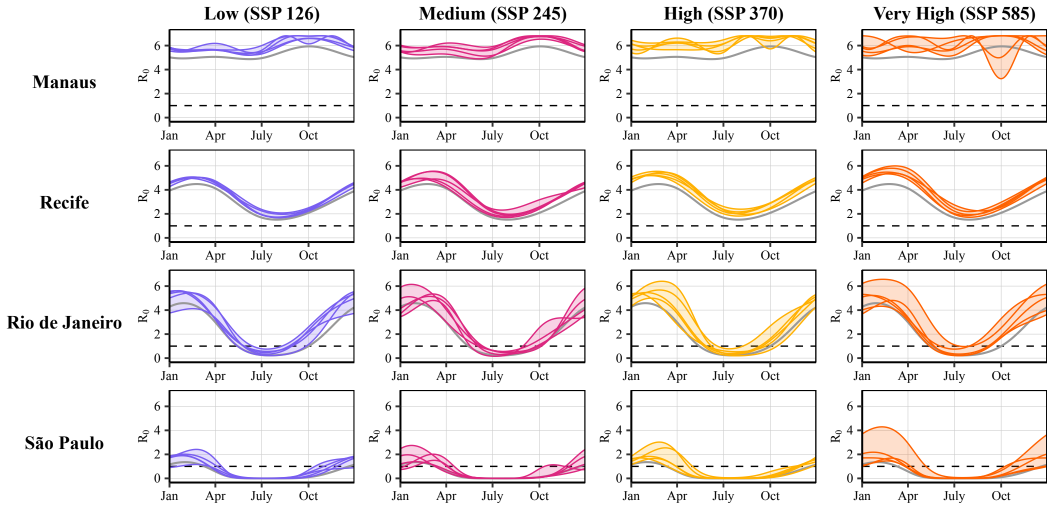

Figure J: **Each panel shows projections of the seasonal temperature in years 2015–2019 for a specific climate change scenario and each city, demonstrating year-to-year heterogeneity in projected risk. Grey lines corresponding to the historical baseline are plotted for comparison. Ribbons were added to highlight the vertical spread in the lines for each year.**

8 Basic reproduction number $\mathcal{R}_0$ calculation

We use the next generation method to derive the basic reproduction number ($\mathcal{R}_0^{NGM}$) [4–6]. The infected subsystem from the equations for this model are as follows:

$$\frac{dL_h}{dt} = a(T)\cdot\pi_{mh}(T)\cdot\frac{I_m}{N_h}\cdot S_h - \sigma_h\cdot L_h - \mu_h\cdot L_h \tag{S2a}$$

$$\frac{dI_h}{dt} = \sigma_h\cdot L_h - \gamma\cdot I_h - \mu_h\cdot I_h \tag{S2b}$$

$$\frac{dL_m}{dt} = a(T)\cdot\pi_{hm}(T)\cdot\frac{I_h}{N_h}\cdot S_m - (\sigma_m(T)+\mu_m(T))\cdot L_m \tag{S2c}$$

$$\frac{dI_m}{dt} = \sigma_m(T)\cdot L_m - \mu_m(T)\cdot I_m \tag{S2d}$$

We decompose the right-hand side of these equations as $\mathcal{F} - \mathcal{V}$, where $\mathcal{F}$ and $\mathcal{V}$ are as follows:

$$\mathcal{F} = \begin{bmatrix} a(T)\cdot\pi_{mh}(T)\cdot\frac{I_m}{N_h}\cdot S_h \\ 0 \\ a(T)\cdot\pi_{hm}(T)\cdot\frac{I_h}{N_h}\cdot S_m \\ 0 \end{bmatrix}, \tag{S3a}$$

$$\mathcal{V} = \begin{bmatrix} (\sigma_h+\mu_h)\cdot L_h \\ (\gamma+\mu_h)\cdot I_h - \sigma_h\cdot L_h \\ (\sigma_m(T)+\mu_m(T))\cdot L_m \\ \mu_m(T)\cdot I_m - \sigma_m(T)\cdot L_m \end{bmatrix}. \tag{S3b}$$

From these, the Jacbobian matrices F of $\mathcal{F}$ and V of $\mathcal{V}$ can be obtained:

$$F = \begin{bmatrix} 0 & 0 & 0 & a(T)\cdot\pi_{mh}(T) \\ 0 & 0 & 0 & 0 \\ 0 & a(T)\cdot\pi_{hm}(T)\cdot N_m/N_h & 0 & 0 \\ 0 & 0 & 0 & 0 \end{bmatrix}, \tag{S4a}$$

$$V = \begin{bmatrix} (\sigma_h+\mu_h) & 0 & 0 & 0 \\ -\sigma_h & (\gamma+\mu_h) & 0 & 0 \\ 0 & 0 & (\sigma_m(T)+\mu_m(T)) & 0 \\ 0 & 0 & -\sigma_m(T) & \mu_m(T) \end{bmatrix} \tag{S4b}$$

Therefore, the next generation matrix is given by

$$FV^{-1} = \begin{bmatrix} 0 & 0 & \frac{a(T)\cdot\pi_{mh}(T)\cdot\sigma_m(T)}{\mu_m(T)(\sigma_m(T)+\mu_m(T))} & \frac{a(T)\cdot\pi_{mh}(T)}{\mu_m(T)} \\ 0 & 0 & 0 & 0 \\ \frac{a(T)\cdot N_m\cdot\pi_{hm}(T)\cdot\sigma_h}{N_h\cdot(\sigma_h+\mu_h)(\gamma+\mu_h)} & \frac{a(T)\cdot N_m\cdot\pi_{hm}(T)}{N_h\cdot(\gamma+\mu_h)} & 0 & 0 \\ 0 & 0 & 0 & 0 \end{bmatrix}, \tag{S5}$$

which has the following characteristic equation,

$$0 = \lambda^4 - \lambda^2 \left(\frac{a(T) \cdot N_m \cdot \pi_{mh} \cdot \sigma_m(T)}{\mu_m(T) \cdot N_h \cdot (\sigma_m(T) + \mu_m(T))} \right) \left(\frac{a(T) \cdot \pi_{hm}(T) \cdot \sigma_h}{(\sigma_h + \mu_h)(\gamma + \mu_h)} \right). \tag{S6}$$

Therefore,

$$\mathcal{R}_0^{NGM} = \sqrt{\frac{a(T) \cdot \pi_{mh}(T) \cdot \sigma_m(T)}{\mu_m(T)(\sigma_m(T) + \mu_m(T))} \cdot \frac{a(T) \cdot \pi_{hm}(T) \cdot \sigma_h}{(\sigma_h + \mu_h)(\gamma + \mu_h)} \cdot \frac{N_m}{N_h}} \tag{S7}$$

References

[1] Huber JH, Childs ML, Caldwell JM, Mordecai EA. Seasonal temperature variation influences climate suitability for dengue, chikungunya, and Zika transmission. PLOS Neglected Tropical Diseases. 2018;12(5):e0006451.

[2] Mordecai EA, Cohen JM, Evans MV, Gudapati P, Johnson LR, Lippi CA, et al. Detecting the impact of temperature on transmission of Zika, dengue, and chikungunya using mechanistic models. PLOS Neglected Tropical Diseases. 2017;11(4):e0005568.

[3] Tesla B, Demakovsky LR, Mordecai EA, Ryan SJ, Bonds MH, Ngonghala CN, et al. Temperature drives Zika virus transmission: evidence from empirical and mathematical models. Proceedings of the Royal Society B. 2018;285(1884):20180795.

[4] Diekmann O, Heesterbeek JAPP, Metz JAJJ. On the definition and the computation of the basic reproduction ratio R0 in models for infectious diseases in heterogeneous populations. Journal of Mathematical Biology. 1990;28(4):365-82.

[5] Van Den Driessche P, Watmough J. Reproduction numbers and sub-threshold endemic equilibria for compartmental models of disease transmission. Mathematical Biosciences. 2002;180:29-48.

[6] Diekmann O, Heesterbeek JAP, Roberts MG. The construction of next-generation matrices for compartmental epidemic models. Journal of the Royal Society, Interface. 2010;7(47):873-85.
